# Case Report: A crushing sensation—a rare case of mediastinal germ cell tumor causing cardiac tamponade

**DOI:** 10.3389/fcvm.2024.1539900

**Published:** 2025-01-20

**Authors:** Kevin S. Tang, Emin Zargarian, Pranav M. Patel

**Affiliations:** ^1^ Department of Internal Medicine, University of California Irvine Health, Orange, CA, United States; ^2^ Mary & Steve Wen Cardiovascular Division, Department of Internal Medicine, University of California Irvine Health, Irvine, CA, United States

**Keywords:** cardiac tamponade, mediastinal tumor, cardiac critical care, germ cell tumor (GCT), pericardial effusion

## Abstract

Cardiac tamponade often presents as external compression of the heart by pericardial fluid, leading to hemodynamic instability, but it can rarely be caused by compression by a solid mass. In this report, we present a case of tamponade-like physiology resulting from a large mediastinal tumor. This is an unusual phenomenon that is rarely described in the literature, and its optimal management remains controversial. This report reviews the clinical considerations for this rare pathophysiology; definitive therapy requires the involvement of a multidisciplinary approach for hemodynamic optimization and mass removal.

## Introduction

Cardiac tamponade is a potentially fatal condition caused by an external compression of the heart chambers, most often resulting from an accumulation of fluid in the pericardial space (1, 2). Classic etiologies of pericardial effusion leading to cardiac tamponade include traumatic hemorrhage, aortic dissection extending into the pericardium, or slow-growing effusions secondary to infection, malignancy, or autoimmune disease, among others (2, 3). Extrinsic cardiac compression by mass effect is an exceedingly rare cause of cardiac tamponade and has been represented in the literature only by disparate case reports (4–6). Physiologic considerations may become further complicated when a large mediastinal tumor copresents with a large pericardial effusion, the removal of which may paradoxically worsen tamponade physiology (4). Particularly in patients who require intensive care unit (ICU) level of care, few guidelines exist on the optimal management of hemodynamic and respiratory instabilities. In this report, we present a case of mediastinal germ cell tumor and concomitant pericardial effusion in an otherwise healthy young adult man, presenting as impending tamponade with a subsequent worsening of tamponade physiology post-pericardiocentesis. We also present a clinical discussion centered around definitive management during the patient’s prolonged and complex hospital course.

## Case summary

A 25-year-old man presented to the Emergency Department (ED) with a chief complaint of abdominal pain. He had been evaluated for the same symptoms several months before in Mexico, but the workup at that time was reported to be unremarkable. He was otherwise healthy without any significant past medical history. On presentation, he was noted to be tachycardic with a heart rate of 140 bpm and hypotensive to 99/58 mmHg. Physical examination revealed significant jugular venous distention (JVD) and distant heart sounds. Pulsus paradoxus was not assessed in the ED. Results of pulmonary and abdominal examinations were otherwise within normal limits. An electrocardiogram was consistent with sinus tachycardia.

**Figure 1:**
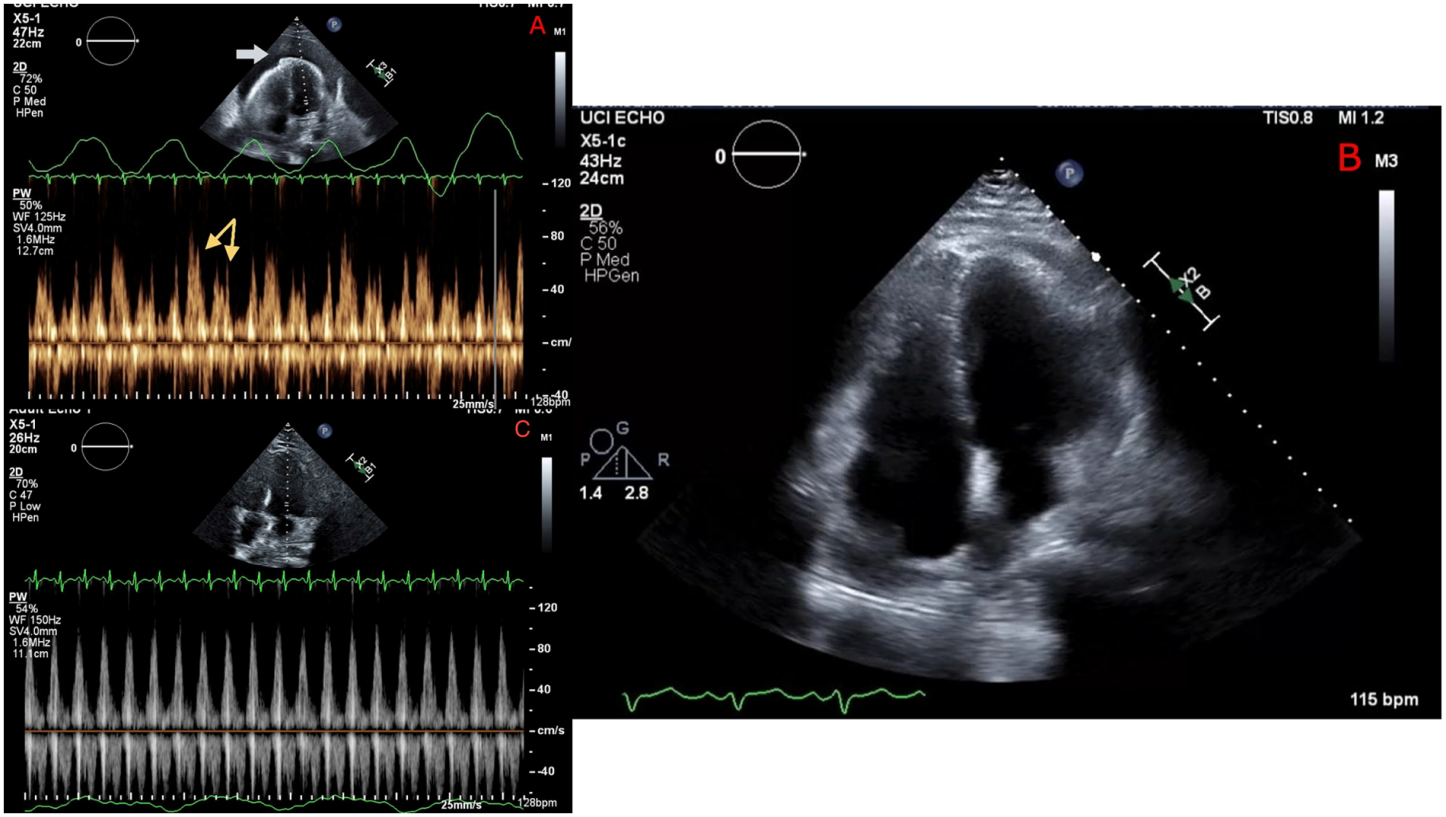
Echocardiography before pericardiocentesis **(A)** showing a large pericardial effusion (white arrow), with Doppler revealing significant respiratory variation of mitral inflow velocity (yellow arrows) consistent with tamponade. Repeat echocardiography immediately following pericardiocentesis **(B)** showing resolution of pericardial effusion and **(C)** no significant respiratory mitral valve inflow variation.

An echocardiogram demonstrated a large pericardial effusion, inversion of the right ventricular (RV) wall in early diastole, ventricular interdependence, significant respiratory variation in mitral and tricuspid inflow velocities, and a plethoric inferior vena cava (IVC), which were all concerning for tamponade physiology. A chest computed tomography (CT) was remarkable for a large anterior mediastinal mass abutting the pericardium, which was deemed to most likely represent a germ cell tumor, although lymphoma could not be definitively excluded on imaging alone. The patient received emergent pericardiocentesis and drain placement with evacuation of 650 ml of sanguineous fluid, successfully reducing pericardial pressure from 28 to 5 mmHg. A repeat echocardiogram showed a resolution of the pericardial effusion and a resolution of RV wall inversion and respiratory inflow velocities (Figure 1).

**Figure 2:**
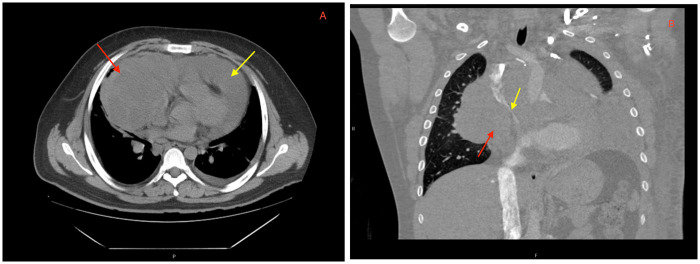
CT imaging showing a **(A)** large mediastinal mass (red arrow) and pericardial effusion (yellow arrow) on presentation. Repeat CT angiography post-pericardiocentesis **(B)** redemonstrating a mediastinal mass (red arrow) abutting the right atrium and ventricle with significant narrowing of the superior vena cava (yellow arrow).

**Figure 3:**
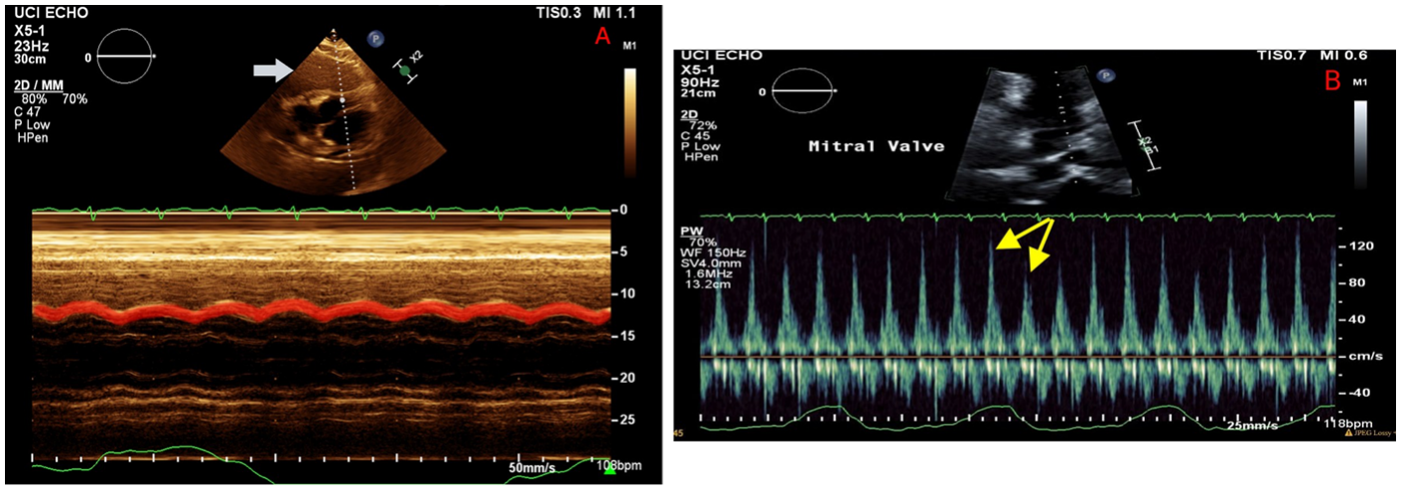
Echocardiography on hospital day 2 illustrating a large mass abutting the right ventricle **(A**, white arrow**)**, with M mode demonstrating a diastolic collapse of the right ventricular wall (red highlight). Mitral valve Doppler **(B)** showing persistent respiratory variation of mitral valve inflow (yellow arrows), consistent with recurrent tamponade physiology despite resolution of the pericardial effusion.

On hospital day 2, the patient developed worsening tachycardia and respiratory distress. Physical examination noted a tachycardia rate of 130 bpm with clear heart sounds and a blood pressure of 101/68 mmHg; JVD was present, and pulsus paradoxus was appreciated with a respiratory variation of systolic blood pressure (SBP) >20 mmHg. Repeat CT showed interval enlargement of the mediastinal mass with right atrial and RV compression, significant narrowing of the main pulmonary arteries, and compression of the superior vena cava (Figure 2). Repeat echocardiogram showed minimal reaccumulation of pericardial fluid but redemonstrated the large mediastinal mass, with compression of the right ventricle causing significant respiratory inflow variability in the mitral valve (40%), tricuspid valve (30%), and aortic valve (20%) spectral Doppler velocities with septal bounce that was concerning for tamponade physiology (Figure 3). He was resuscitated with 1 L of lactated Ringer's solution and brought to the ICU for stabilization. Conservative measures were unsuccessful in reversing his respiratory distress, which was thought to be secondary to a combination of imminent tamponade, pulmonary artery compression, and significant left lung atelectasis due to compression from the space-occupying lesion. Increasing oxygen requirements and respiratory fatigue prompted intubation and mechanical ventilation. The decision-making process involved cardiothoracic surgery, which deemed the patient to be a poor surgical candidate because of the unacceptable risk of cardiopulmonary collapse with general anesthesia.

Instead, thoracic surgery involved the performance of mediastinoscopy and biopsy on the patient via the Chamberlain procedure, which on pathology revealed a non-seminomatous germ cell tumor. Following a discussion with the oncology department, the patient was started on cisplatin, etoposide, and ifosfamide (VIP) chemotherapy, ultimately completing four cycles during his hospitalization. This treatment appeared to be successful in decreasing the size of the tumor, as subsequent echocardiograms noted improvement and then resolution of extrinsic cardiac compression by hospital day 45. Unfortunately, the remainder of his hospital course was riddled with multiple complications, including septic shock, acute respiratory distress syndrome, ventilator-associated pneumonia, bilateral lower- and upper-extremity deep venous thromboses, left-ventricle apical thrombus, and acute renal failure. He also developed significant pancytopenia, which was deemed secondary to the myelosuppressive effects of his chemotherapy. He ultimately succumbed to his illness and died on hospital day 142 (Table 1).

**Table 1:** Hospitalization timeline.

Hospital day	Events
1	Presentation with abdominal pain and Beck's triad. Successful emergent pericardiocentesis with drain placement.
2	Worsening respiratory distress with pulsus paradoxus and increased right ventricular compression on echocardiography. Intubated and upgraded to the intensive care unit.
5	Successful mediastinal mass biopsy with cardiothoracic surgery.
12	Pathology confirmation of a non-seminomatous germ cell tumor.
13	Initiation of VIP chemotherapy.
18	Given extended ventilator dependence, tracheostomy was performed by the trauma surgery department.
25	Patient found to have non-occlusive pulmonary embolism in the right lower lobe and bilateral lower-extremity deep venous thromboses.
45	Repeat echocardiography showing a decrease in the size of the mediastinal mass, now without extrinsic cardiac compression.
60	Left ventricular apical thrombus found on repeat echocardiography.
68	Tunneled dialysis catheter placed for persistent renal failure requiring hemodialysis.
134	Patient diagnosed with ventilator-associated *Staphylococcus aureus* pneumonia.
142	Patient transferred to inpatient hospice service and expired.

## Discussion

Cardiac tamponade is most often precipitated by an accumulation of fluid in the pericardial space, and thus, the gold standard management of tamponade has been timely drainage for hemodynamic improvement (2, 7). The management of tamponade physiology secondary to mass effect, however, is not well defined. In cases of mediastinal tumors, as in our patient, the mechanism of tamponade may be mixed or multifactorial due to both external compression by the tumor and accumulation of a malignant effusion.

The differential diagnosis for mediastinal masses is broad and encompasses a wide range of childhood and adult disorders. Of the potential etiologies, thymomas, neurogenic tumors, and foregut cysts comprise the majority (60%) of all mediastinal masses (8). Germ cell tumors, neurogenic tumors, and benign cysts are most commonly present in childhood, while thyroid masses and lymphomas constitute the majority of adult presentations (8, 9). Accurate diagnosis necessitates advanced imaging with CT or MRI, followed by biopsy and pathologic evaluation.

Large mediastinal masses with consequent pericardial effusions and concern for impending tamponade require a cautious multidisciplinary approach while pending diagnostic imaging and pathology. While not specific to cardiac tamponade, pulsus paradoxus may be present on physical examination and is caused by an increase in preload during inspiration that pushes the interventricular septum into the left ventricular space, thus transiently decreasing left ventricular volume and cardiac output (6). There are few reports on the optimal immediate management for optimization of hemodynamics, but some have suggested that rapid drainage of pericardial fluid may paradoxically worsen obstructive shock by removing the fluid buffer between the tumor and the ventricular wall (4). These patients remain heavily preload-dependent, and adequate fluid resuscitation is essential to equalize pericardial and intraventricular pressures and maintain hemodynamic stability (4, 5, 10). Our patient's clinical course emphasizes these key considerations with the physical examination findings, such as pulsus paradoxus, that would be expected with recurrent tamponade. In patients with similar presentations consisting of a large mediastinal tumor and pericardial effusion, consideration should be given to partial or staged pericardiocentesis to preserve the fluid buffer and allow additional time for histopathological diagnosis and definitive management.

In patients requiring intubation, invasive positive pressure ventilation should be pursued with caution as it may precipitate mediastinal mass syndrome. This life-threatening syndrome is characterized by cardiorespiratory decompensation on the initiation of positive pressure ventilation and is thought to be caused by an acute decrease in venous return in the setting of a preload-starved state due to tumor compression, leading to significantly impaired cardiac output and worsening obstructive shock (5, 11). Mediastinal mass syndrome may be further worsened by tracheal or bronchial compression, which may be suggested by orthopnea or dysphagia from concomitant esophageal narrowing (11). In cases where mediastinal mass syndrome leads to cardiopulmonary arrest, extracorporeal life support (ECLS) should be considered as early as possible. Chest compressions may be fatally ineffective in the presence of a compressive mediastinal mass, and downward pressure would push the tumor further against the right ventricle, thereby preventing ventricular filling and any meaningful cardiopulmonary circulation (5). While our patient did not develop the devastating consequences of mediastinal mass syndrome following intubation and was able to be stabilized in the ICU, ECLS would have been an important consideration if his hemodynamics had worsened further. This would have also allowed further consideration of aggressive operative intervention. Immediate tumor resection was deferred by the cardiothoracic surgery department due to concerns of increased airway compression and mortality with induction in the setting of a large mediastinal mass effect (12), but this risk would likely have been mitigated in a patient on ECLS.

In the absence of acute cardiopulmonary decompensation, biopsy confirmation of malignancy should be followed by a multidisciplinary approach to mass reduction or removal; definitive treatment involves surgical resection or chemotherapy depending on the underlying etiology (13). For mediastinal germ cell tumors, definitive treatment is characterized by cisplatin-based chemotherapy followed by surgical resection of any residual tissue (14). Our patient was planned for eventual surgical resection with cardiothoracic surgery, but unfortunately never improved sufficiently to be discharged and followed up for adjuvant surgical management. Special attention should also be paid to the management of malignancy-associated hypercoagulability. As evidenced by our patient's hospital course, the development of multiple instances of venous thromboembolism (VTE) is a significant risk with potentially devastating consequences, particularly in association with the maintenance of central venous catheters and acute illness immobility (15). A Khorana score should be calculated to promptly risk-stratify patients and identify those in whom it may be prudent to initiate early VTE prophylaxis (16, 17). Although our patient was initiated on appropriate VTE prophylaxis immediately after the Chamberlain procedure on hospital day 3, he still developed several VTE-associated complications, probably because of persistently severe illness and prolonged in-hospital immobility.

Cardiac tamponade secondary to mediastinal tumor is a rare and complex condition that requires a nuanced approach with additional clinical considerations compared with tamponade secondary to pericardial fluid alone. Partial or staged drainage of tumor-associated pericardial fluid should be pursued by an experienced provider to avoid a paradoxical worsening of hemodynamics from massive external compression. Management should be multidisciplinary and pursued in a specialized medical center with access to cardiothoracic surgery to aid in prompt tissue diagnosis and consideration of ECLS and operative intervention in cases of hemodynamic collapse.

## Data Availability

The original contributions presented in the study are included in the article/Supplementary Material, further inquiries can be directed to the corresponding author.
